# Ionic Strength Impacts the Physical Properties of Agarose Hydrogels

**DOI:** 10.3390/gels10020094

**Published:** 2024-01-25

**Authors:** Pasquale Sacco, Francesco Piazza, Eleonora Marsich, Michela Abrami, Mario Grassi, Ivan Donati

**Affiliations:** 1 Department of Life Sciences, University of Trieste, Via Licio Giorgieri 5, I-34127 Trieste, Italy; francesco.piazza2@phd.units.it (F.P.); idonati@units.it (I.D.); 2 Department of Medicine, Surgery and Health Sciences, University of Trieste, Piazza dell’Ospitale 1, I-34129 Trieste, Italy; emarsich@units.it; 3 Department of Engineering and Architecture, University of Trieste, Via A. Valerio 6/1, I-34127 Trieste, Italy; michela.abrami@dia.units.it (M.A.); mario.grassi@dia.units.it (M.G.)

**Keywords:** agarose, hydrogel, ionic strength, structuring of the water, mechanical properties

## Abstract

Agarose is a natural polysaccharide known for its ability to form thermoreversible hydrogels. While the effects of curing temperature and polysaccharide concentration on mechanical properties have been discussed in the literature, the role of ionic strength has been less studied. In the present manuscript, we investigate the effects of supporting salt concentration and the role of cation (i.e. Na^+^ or Li^+^, neighbors in the Hofmeister series), on the setting and performance of agarose hydrogels. Compressive and rheological measurements show that the supporting salts reduce the immediate elastic response of agarose hydrogels, with Li^+^ showing a stronger effect than Na^+^ at high ionic strength, while they significantly increase the extent of linear stress-strain response (i.e., linear elasticity). The presence of increasing amounts of added supporting salt also leads to a reduction in hysteresis during mechanical deformation due to loading and unloading cycles, which is more pronounced with Li^+^ than with Na^+^. The combination of rheological measurements and NMR relaxometry shows a mesh size in agarose hydrogels in the order of 6–17 nm, with a thickness of the water layer bound to the biopolymer of about 3 nm. Of note, the different structuring of the water within the hydrogel network due to the different alkali seems to play a role for the final performance of the hydrogels.

## 1. Introduction

Agarose is a neutral (uncharged) polysaccharide obtained from marine red algae. Chemically, agarose consists of an alternation of D-galactose and 3,6-anhydro-L-galactose monomers (repeating unit) linked by α-(1→3) and *β*-(1→4) glycosidic bonds [1]. Unlike natural or modified polycations such as chitosans and their derivatives [2,3,4,5,6], or polyanions such as alginate [7], which in most cases require the use of specific cross-linking agents that allow gelation, agarose follows a temperature-assisted sol-gel transition after the formation of hydrogen bonds. Agarose is insoluble when dispersed in water at room temperature. At high temperatures, agarose dissolves forming random coil structures. Upon cooling, helical motifs and bundles form that allow liquid-liquid phase separation (gelation) [8,9,10,11]. With respect to helical motifs, it was found that they are double- and single-stranded α-helix conformation in hydrogels of native agarose, while unpaired *β*-strands in carboxylated-modified networks [12].

To date, there are two main theories explaining the gelation mechanism of agarose during cooling: spinodal decomposition [9,10,11] or nucleation and growth [13]. A pivotal difference between the two is the existence of an induction time before phase separation originates. If phase separation occurs through a nucleation and growth mechanism, an induction time should not only exist, but also conform to the rules of nucleation theory [13]. In this view, it has been demonstrated that the gelation process of agarose occurs through three different stages, entailing (1) induction, (2) gelation and (3) pseudoequilibrium stage. The topology and, therefore, physical and mechanical properties of agarose-based hydrogels are strictly dependent on temperature and, more broadly, the rate of quenching of agarose solutions or thermal history after gelation [13,14,15]. Regarding quenching, some of us have recently reported that the physical properties of agarose hydrogels are strongly influenced by the cooling rate of agarose solutions after autoclaving, leading to fundamental effects on the architecture of the hydrogel network, the mechanical response of both the surface and the bulk, and the response of cells when cultured on them [14]. In addition to temperature, molecular weight or concentration of agarose could have an effect on the gelation process [16,17]. For example, a low agarose concentration allows biopolymer chains to aggregate in a helical conformation and subsequently form clusters. As the polymer concentration increases, a sol-gel transition occurs in which the clusters combine via fibrillar bundles of different compositions to form a hydrogel [18,19]. It is interesting to note that agarose-based hydrogels show different mesh size, ranging in the order of hundreds of nanometers, depending on its concentration [20,21]. Furthermore, the degree and pattern of residual methylation has been shown to play a role in the mechanical response of hydrogels from agaroses of different origins, especially when the nonlinear stress-strain region is considered [22].

Agarose-based hydrogels were widely used as a platform for biomedical applications due to their known biocompatibility [1,23,24,25,26,27,28,29,30,31]. In this context, it is important to understand how the presence of salts at different concentration affects the mechanical response of agarose hydrogels. This is particularly important considering Hofmeister effects on the mechanical properties of hydrogels. More in detail, the addition of “chaotropic” ions will gain more sensitive and softer hydrogels, while the “kosmotropic” ions will induce the production of stiff hydrogels with less sensitivity [32]. Though agarose is a neutral macromolecule, residuals of agaropectins with negatively charged sulfated groups are found in commercial samples, with their polyelectrolyte character complicating the response of the network. It has been shown that when sulfate anions (Na^+^ as counterion) are added to agarose 1% *w*/*w* with low sulfated content, i.e., < 0.25%, varying the ionic strength of the system, the anion affects the final hydrogel structure by negatively influencing the coil-helix transition and by hampering the helix-helix interactions [33]. A closer examination of the hydration properties has shown that the addition of sodium sulfate weakens the agarose-water interactions, thus facilitating the easy release of water from the hydrogel network and increasing agarose hydrophobicity. Overall, the results reported in this paper indicate that increasing the ionic strength while varying the sodium sulfate content reduces the structuring of the water throughout the hydrogel. It is interesting to note that in a previous study it was shown that the mechanical properties of agarose hydrogels at 2% *w*/*w* are almost independent when prepared in the presence of alkali metal salts such as NaCl or KCl and analyzed for *T* < 65 °C up to a ionic strength of 4 M [34]. The authors attributed this behavior to the naturally stable macromolecular structure of the agarose network, which is almost insensitive to the addition of ions.

Considering that ionic strength and water organization in the hydrogel network play a role in the final hydrogel structure, here we prepare 1% *w*/*V* agarose hydrogels in the presence of different amounts of alkali metal salts (NaCl and LiCl) and investigate the effects of ionic strength on the mechanical properties at small/large deformations, water magnetic relaxation time and payload diffusion.

## 2. Results and Discussion

**Figure 1:**
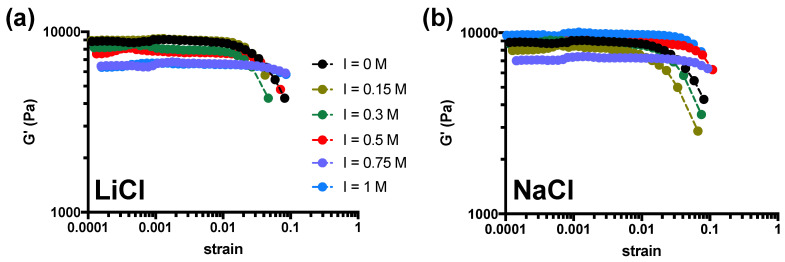
Long stress sweep experiments on agarose hydrogels supplemented with different amounts of alkali metal salts LiCl (**a**) or NaCl (**b**) to vary ionic strength, *I*. Data are reported as a dependence of the elastic modulus, *G′*, on the applied strain at a constant frequency of 1 Hz. Dashed lines are drawn to guide the eye. Experimental conditions: [agarose] = 1% *w*/*V*; *I* = 0–1 M; *T* measurement = 37 °C.

We have investigated the effects of ionic strength, *I*, generated by different alkali metal salts on the mechanical properties of hydrogels composed of a commercial agarose sample with low agaropectin content [22]. Agarose powder was dispersed in the presence of two monovalent salts, namely LiCl and NaCl, to vary the final ionic strength in the range 0–1 M. After autoclaving the mixtures at 121 °C and immediately quenching at room temperature, the agarose hydrogels were equilibrated at 37 °C for another 24 h [13] and analyzed by stress sweep experiments at 1 Hz. Regardless of the monovalent salt or concentrations used, agarose hydrogels show similar behavior, with the elastic modulus, *G′*, being independent on the applied strain at low deformations, while gradually decreasing at large deformations, i.e., showing strain-softening behavior consistent with previous results using PBS buffer as medium (Figure 1) [14]. For all samples analyzed, *G″* is at least one order of magnitude lower than *G′*.

**Figure 2:**
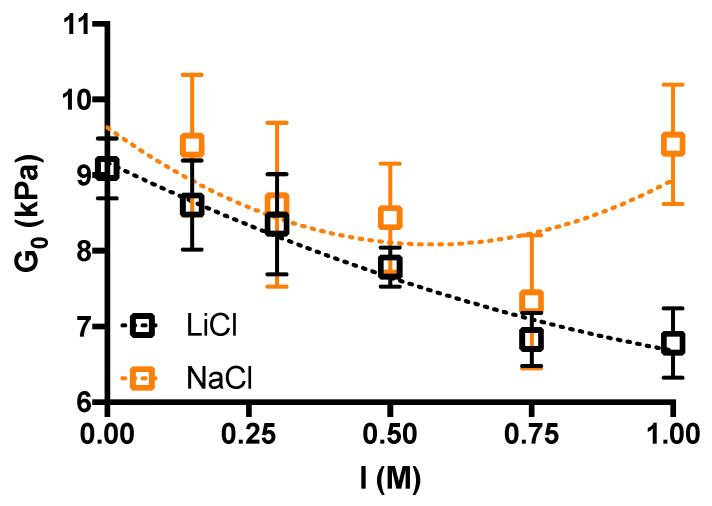
The effect of ionic strength, *I*, on the elastic response of agarose hydrogels, $G_{0}$ , under oscillatory mechanical stimulation. $G_{0}$ values are calculated from Equation (1) in the main manuscript. Data are displayed as mean ± sd. Dotted lines are drawn to guide the eye. Experimental conditions: [agarose] = 1% *w*/*V*; *I* = 0–1 M; *T* measurement = 37 °C.

The dependence of the elastic modulus *G′* for strain values tending towards zero indicates a decrease in the elastic response for both LiCl- and NaCl-supplemented hydrogels for a total ionic strength of up to 0.75 M. A further increase in ionic strength does not show a variation in the case of LiCl, while an increase in *G′* is observed for NaCl. The data of the long stress sweep were analyzed with respect to the stress-strain relationship and modelled according to Equation (1) to provide information about $G_{\gamma \to 0}=G_{0}$

(1) $$\sigma =\frac{G_{0}}{1+b\gamma }\gamma \;$$

where *b* is a fitting parameter, σ the applied stress and $G_{0}$ the shear modulus for strain →0 [14,22]. Figure 2 shows the trend of $G_{0}$ as a function of ionic strength when both monovalent salts are considered. 

Interestingly, the hydrogels supplemented with LiCl show an almost monotonic decrease up to ~25% in the elastic response upon increasing the ionic strength, whereas for the hydrogels supplemented with NaCl, a decrease in $G_{0}$ is followed by an upturn of the curve. From these results, it can be concluded that the effect of NaCl is akin to LiCl on $G_{0}$ up to *I* = 0.75, whereas diverging for *I* = 1 M. As detailed in Section 4 (Average mesh size determination), given $G_{0}$ values, Flory’s and equivalent network theories allow computing the average network mesh size, $\bar{\xi }$ [35], which is around 10 nm for hydrogels of both alkali metals investigated, and almost independent on *I*.

Next, we calculated the critical strain, $\gamma_{c}$ , which marks the onset of the non-linear behavior, that can be arbitrarily determined as Equation (2) [22]. Table 1 summarizes the results of $G_{0}$ and $\gamma_{c}$ obtained from Equations (1) and (2). It is interesting to note that the critical strain is much more influenced by the addition of monovalent salts than $G_{0}$ , with a general extension of linear elasticity upon increasing LiCl or NaCl.

(2) $$\gamma_{crit}:\;\;\;\;\;\frac{\sigma }{G_{0}\cdot \gamma }=0.95\;\;$$

**Figure 3:**
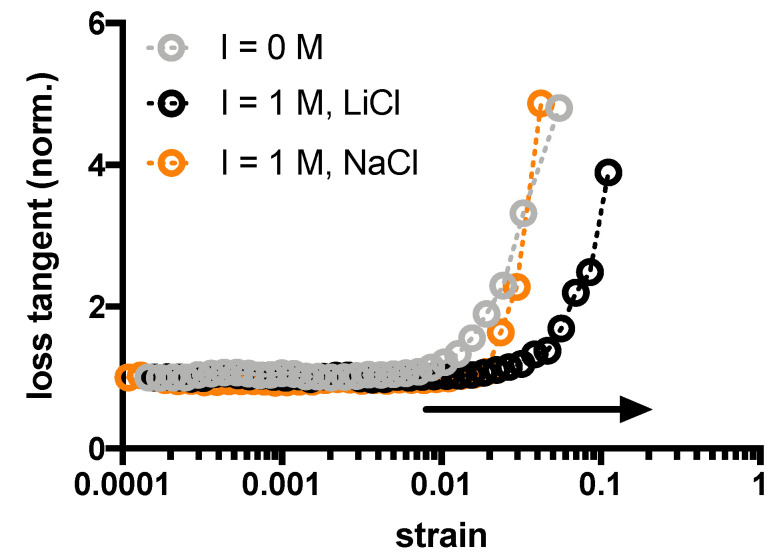
Dependence of loss tangent recorded at 1 Hz as a function of applied strain for agarose hydrogels prepared in the presence of alkali metal salts or pure deionized water. Dotted lines are drawn to guide the eye. Experimental conditions: [agarose] = 1% *w*/*V*; *I* = 0 or 1 M; *T* measurement = 37 °C.

The effect of ionic strength on the viscous contribution upon increasing the applied strain was determined in terms of loss tangent, that is tanδ=G″G′ (Figure 3). For the sake of comparison, the effect of *I* = 1 M for both monovalent salts has been compared with agarose hydrogels that have been assembled in deionized water. In both cases, the addition of monovalent salts promotes a shift in the raise of loss tangent, albeit to a different extent: the effect of LiCl overcomes the one by NaCl. Taken together, the rheological results suggest that the mechanical behavior of agarose hydrogels depends on both the ionic strength and the type of alkali metal used to regulate the ionic strength, in particular for the extent of elasticity and viscous response, while there is only a slight effect on the shear modulus almost up to *I* = 0.75 M. In addition, the presence of supporting salts does not modify the gel setting temperature, which remains around 34 °C for all salt concentrations tested as demonstrated by hard shaking method. Furthermore, once the hydrogel is set in the presence of supporting salt, its removal by extensive washing in deionized water has no effect on the mechanical performance (Figure S1 in Supplementary Materials).

**Figure 4:**
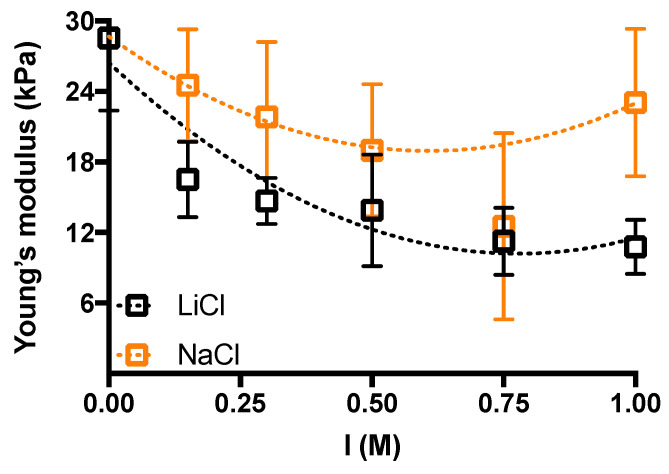
The effect of ionic strength, *I*, on the Young’s modulus of agarose hydrogels under uniaxial compression. Young’s modulus values are calculated in the linear stress-strain region (1–4%). Data are displayed as mean ± sd. Dotted lines are drawn to guide the eye. Experimental conditions: [agarose] = 1% *w*/*V*; *I* = 0–1 M; *T* measurement = 25 °C.

The effect of ionic strength on the mechanical response of agarose hydrogels has been analyzed also by uniaxial compression measurements. Linear stress-strain region in the range 1–4% has been considered in order to determine the Young’s modulus for agarose hydrogels obtained in the presence of monovalent salts at different concentration (Figure 4). 

In nice agreement with the rheological data, we observed a decrease in the elastic response of the hydrogel when the amount of monovalent salt added was increased up to *I* = 0.75 M, albeit much more oscillating within experimental error in the case of NaCl. It is interesting to note that the hydrogels supplemented with LiCl show an initial decrease in the Young’s modulus of about 50% for the first addition of supporting salt, to reach a decrease of approx. 60% for the higher *I* tested, i.e., *I* = 1 M. 

**Figure 5:**
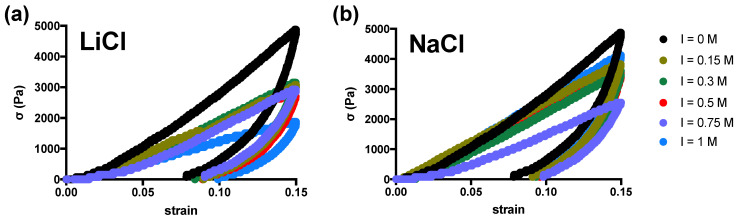
Loading-unloading cycle for agarose hydrogels supplemented with varying amount of alkali metal salts LiCl (**a**) or NaCl (**b**) to regulate the ionic strength. The area delimitated by curves represents the area of hysteresis, that is the energy loss (J/m^3^) by the hydrogel network upon loading/unloading cycle. Experimental conditions: [agarose] = 1% *w*/*V*; I = 0–1 M; T measurement = 25 °C.

Agarose hydrogels were next strained up to 15% of total deformation following a loading-unloading cycle to investigate their response in the non-linear region (Figure 5). 

**Figure 6:**
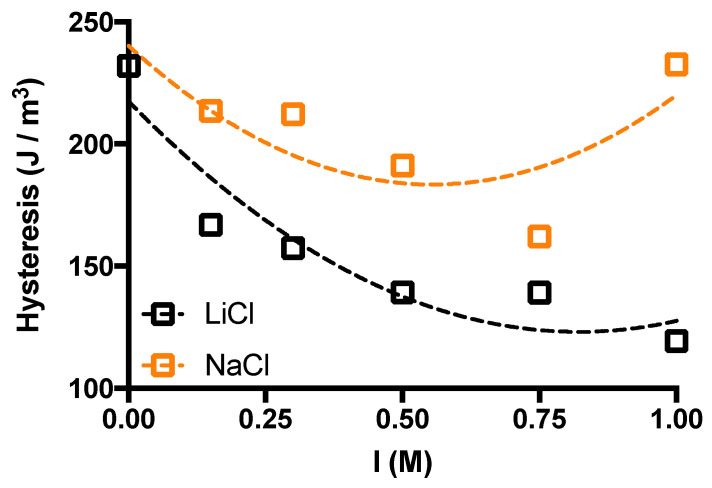
The effect of ionic strength, *I*, on the hysteresis area of agarose hydrogels under uniaxial compression. Loading/unloading cycles have been performed up to 15% of total deformation. Data are displayed as mean of 3–6 hydrogels for each condition. Dashed lines are drawn to guide the eye. Experimental conditions: [agarose] = 1% *w*/*V*; *I* = 0–1 M; *T* measurement = room temperature.

The first consideration that emerges for both monovalent salts used is that the unloading path marks a significant hysteresis area, indicating energy loss, a typical behavior of viscoelastic hydrogels [36]. In the case of LiCl-supplemented hydrogels, there is a significant reduction of the maximum stress that causes a deformation of 15%. This effect is more limited when NaCl supplemented hydrogels are considered. Furthermore, quantification of the energy loss density (hysteresis area) highlights an interesting scenario for both systems analyzed (Figure 6).

We observed a stronger dependence on the ionic strength of the LiCl-supplemented hydrogels than in the NaCl counterparts. The decrease in hysteresis area follows an increase in I, which is particularly evident in the LiCl-supplemented hydrogels. This parallels the results of long stress sweep experiments, where the higher $\gamma_{c}$ for hydrogel samples with higher salt concentrations—that is 1 M—can be related to the higher elastic energy stored by the hydrogel in the linear stress-strain region. Taking the rheological results and the uniaxial compression results together, the influence of the type of cation on the response of the hydrogels after mechanical stimulation traces back to the different structuring of the water within the hydrogel network due to different alkali cations [37], given the more “chaotropic” nature of Li^+^ with respect to Na^+^.

**Figure 7:**
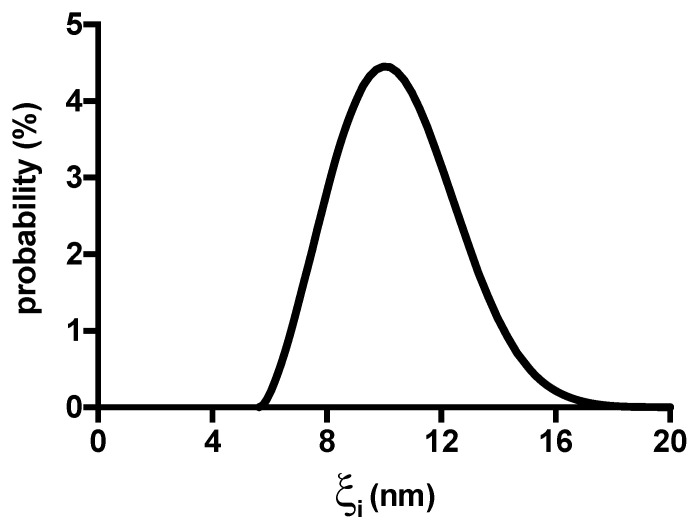
Mesh size distribution of agarose hydrogel referring to *I*(M) = 0 sample.

Low-field NMR allows inferring the distribution of mesh size of polysaccharide-based hydrogels [38,39]. Indeed, following the strategy presented in [40], it is possible determining the continuous relaxation time distribution that can be transformed into the mesh size distribution once the average mesh size of our samples ( $\bar{\xi }\;$ ≈ 10 nm) is determined relying on the Flory theory and on the knowledge of $G_{0}$ values (see Table 1):

(3) $$\xi_{i}=\bar{\xi }\;\frac{\left({\left(\frac{1}{T_{2}}\right)}_{m}-\;\frac{1}{T_{2\mathrm{free}}}\right)}{\left(\frac{1}{T_{2i}}\;-\;\frac{1}{T_{2\mathrm{free}}}\right)}$$

where $T_{2i}$ is the transverse relaxation time of water molecules trapped inside meshes of size $\xi_{i}$ , $T_{2\mathrm{free}}$ is the transverse relaxation time of “free” water (corresponding to water molecules very far from polymeric chains; in our experimental conditions (T = 25 °C and the applied static magnetic field (B_0_) is equal to 0.47 T) the relaxation time of free water is 3020 ms [40,41]) and ${\left(\frac{1}{T_{2}}\right)}_{m}$ is the average value of the relaxation time inverse referring to the whole polymeric network. As our hydrogels did not undergo significant swelling/shrinking in the temperature range here considered (25 °C—LF-NMR test; 37 °C rheological test aimed at G_0_ evaluation), we can safely approximate G_0_ (25 °C) with its value at 37 °C. Figure 7 shows that the mesh size distribution, referring to *I*(M) = 0 sample, spans from about 6 to 17 nm. 

In order to deeply evaluate possible effects of ions on the whole hydrogels structure, we also estimated the thickness (*a*) and the relaxation time (*T_2b_*) of the “bound” water represented by water molecules close to the polymeric chains. At this purpose, the strategy presented in [42] and implemented in the Materials and Methods section, has been considered. This analysis reveals that both $T_{2b}$ and *a* do not significantly vary among all our samples and their average values read $T_{2b}$ = (18 ± 1.4) ms and *a* = (2.9 ± 0.05) nm for the NaCl added hydrogels while for the LiCl added hydrogels their values read $T_{2b}$ = (17 ± 0.8) ms and *a* = (3.0 ± 0.11) nm. Consequently, the inspection of Figure 7 reveals that in the smallest meshes ( $\xi_{i}$ ≈ 6 nm) free water is practically absent as 2*a* ≈ $\bar{\xi }$ . In addition, it is not worthwhile underlying that $T_{2b}$ is about two orders of magnitude lower than $T_{2\mathrm{free}}$ (3020 ms).

**Figure 8:**
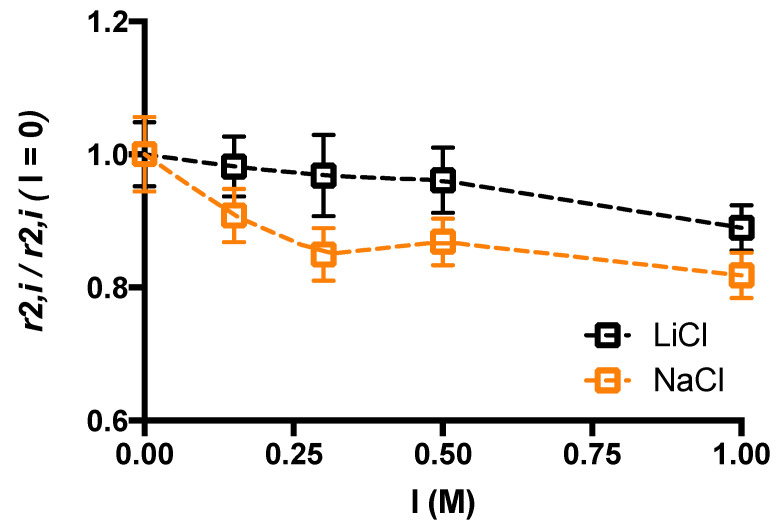
Relative variation of transversal relaxation rate of water molecules as a function of the ionic strength for NaCl and LiCl.

Experimental transversal relaxation rate of water molecules, r2,i =1T2i , can be expressed in terms of the transversal relaxation time of free water, $r_{2,free}$ , and of bound water, $r_{2,bound}$ (Equation (4)):

(4) $$r_{2,i}=X_{\mathrm{free}}r_{2,free}+X_{\mathrm{bound}}r_{2,bound}$$

where $X_{\mathrm{free}}$ and $X_{\mathrm{bound}}$ are the fraction of free and bound water molecules, respectively. Figure 8 shows that the addition of increasing amount of supporting salt, either NaCl or LiCl brings about a moderate decrease in the experimental transverse relaxation rate of water molecules. The presence of the supporting salt changes Equation (4) in the following one (Equation (5)):

(5) $$r_{2,i}=X_{\mathrm{free}}r_{2,free}+X_{\mathrm{bound}}r_{2,\mathrm{bound}}+X_{M^{+}}r_{2,M^{+}}+X_{A^{-}}r_{2,A^{-}}$$

where $X_{M^{+}}$ and $X_{A^{-}}$ are the fraction of water molecules bound to the cation and the anion, respectively, and $r_{2,M^{+}}$ and $r_{2,A^{-}}$ are their relaxation times.

The cation and the anion coordinate water molecules thus affecting the magnetic relaxation of their hydrogens. It is interesting to note that when LiCl is used the relative decrease in $r_{2,i}$ with the supporting salt concentration is lower than in the case of NaCl ascribed to the higher ability of Na^+^ to interact with water (more kosmotropic).

**Figure 9:**
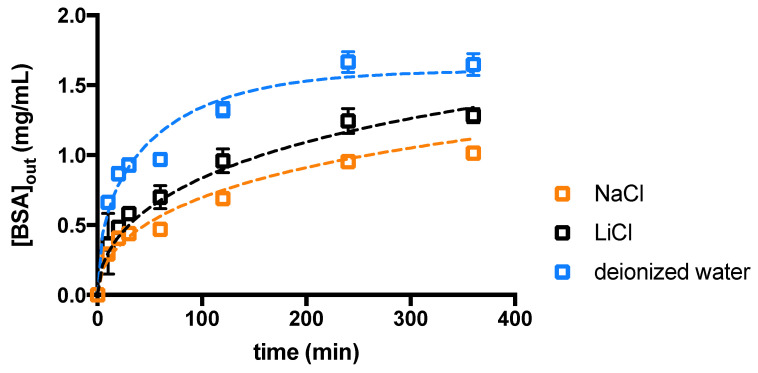
Time-dependent diffusion of BSA measured as concentration in the outer reservoir (BSA_out_) in deionzed water, NaCl 0.5 M and LiCl 0.5 M. Dashed lines represent the best fit of the experimental data using Fick’s second law (Equation (6) in Section 4).

The effect of the supporting salt on agarose hydrogels has also been explored by measuring the diffusion of bovine serum albumin (BSA) protein (Figure 9).

The diffusion coefficients calculated using Fick’s second law are reported in Table S1 in the Supplementary Materials section and are in line with those previously obtained for BSA in polysaccharide-based hydrogels [43]. In addition, it is clear that BSA can diffuse inside the polymeric network of agarose hydrogels as its diameter (≈7.2 nm) approximately corresponds to the smallest mesh size of the mesh size distribution reported in Figure 7. A scheme of BSA diffusion in different conditions is reported in Figures S2 and S3 in Supplementary Materials. The diffusion of BSA in water results approximately 6-times faster than the one in the presence of supporting salt, either NaCl or LiCl. This behaviour contradicts what expected if we simply look to the mean mesh size of the agarose network. Indeed, Figure 4 shows that, following the addition of NaCl and LiCl 0.5 M, respectively, the Young’s modulus of agarose hydrogels decreases. Accordingly, this would reflect in an increase of the mean network mesh size of, about, 9% (NaCl case) and 25% (LiCl case), respectively, in virtue of the inverse relation existing between the Young’s (or shear) modulus and the average mesh size of the polymeric network [40]. Thus, the increment of BSA diffusion coefficient inside agarose hydrogels containing either NaCl or LiCl would be reasonable. Our findings demonstrate that, in this case, the average mesh size variation is much less important than the effect of the electrostatic interactions taking place in salt added agarose hydrogels. Indeed, two different electrostatic interactions have to be considered. The first is connected to the electrostatic repulsion among BSA molecules at pH > pI, as previously reported [44]. The second consists in an entropy-driven enhanced diffusion of BSA due to the counterions of the charged groups of the protein, thus resembling the diffusion of polysaccharide chains in the formation of inhomogeneous alginate hydrogels [7]. The addition of supporting salt, whether NaCl or LiCl, limits both effects thus reducing the diffusion of BSA. Focusing on diffusion experiments in supporting salt, *D* with Li^+^ is slightly higher than with Na^+^, in line with literature reports [44] that described it as due to the balancing of the empirical “law of matching water affinities” rule and of ion polarizability. In addition, the slightly less dense agarose hydrogel network obtained in the presence of Li^+^ could somehow contribute to the higher diffusion coefficient. 

## 3. Conclusions

Agarose forms thermoreversible hydrogels whose mechanical properties can be easily modulated by varying the polysaccharide concentration and the curing temperature. The present work shows that the supporting salt is an efficient and alternative approach to tune the mechanical properties of agarose hydrogels, mostly at high strain values, and payload diffusion. Given the negligible polyelectrolyte character of the agarose used, the role of the supporting salt in the formation of agarose hydrogels relates to the ability to coordinate water molecules rather than significantly impacting on the mesh size of the network. The presence of NaCl or LiCl alters the mechanical properties of the hydrogels without significantly affecting the hydrogel curing temperature and restricts the diffusion of the payload, slowing down the BSA-induced evasive movement due to counterion. The presence of the salts alters sensibly the elasticity of the hydrogels, especially upon compression rather than shear, with Li^+^ showing a stronger effect due to its more chaotropic nature. In addition, once the network is formed, no restructuring takes place when the supporting salt is removed by washing and the mechanical properties are maintained. Since it is known that ionic strength influences the water structure in agarose hydrogel networks and this depends on the overall ability of ions to induce salting in/out phenomena (Hofmeister effects), the present work provides additional information about the amount and type of salts involved and the mechanical response of agarose hydrogels, especially under high loading.

## 4. Materials and Methods

### 4.1. Preparation of Agarose Hydrogels

Agarose LE for electrophoresis was purchased from Euroclone (Italy) (code EMR920500). The physical/chemical characteristics of the agarose are the following: total content of agaropectins (in terms of ashes) = 0.6% *w*/*w*; gelling temperature = 34 °C; rotational viscosity at 60 °C = 14 mPa s; total methylation = 7.4% [22]. Agarose powder was added to deionized water, LiCl (Merck, Darmstadt, Germany) and NaCl (Sigma, St. Louis, MO, USA) solutions prepared at different concentrations (0 M, 0.15 M, 0.3 M, 0.5 M, 0.75 M and 1 M). For rheological and compression tests, agarose was added to the solvents under vigorous stirring to achieve a final concentration of 1% *w*/*V*. The resulting suspensions were autoclaved and the obtained solutions were cast into cylindrical supports and left to cool down at room temperature for 30 min. Finally, the hydrogels were incubated for 24 h at 37 °C under water-saturated conditions to prevent evaporation of the solvent and then analysed. For BSA diffusion test, 2% *w*/*V* agarose and BSA 30 mg/mL solutions were prepared respectively in deionized water, LiCl 0.5 M, and NaCl 0.5 M and then placed in a thermal bath at 42 °C until thermal equilibrium was reached. Equal volumes of agarose and BSA solutions were mixed to achieve a final concentration of 1% *w*/*V* agarose and 15 mg/mL BSA. The mixture was cast into cylindrical supports, left to cool down at room temperature for 30 min and incubated for 24 h at 37 °C under water-saturated conditions to prevent evaporation.

### 4.2. Compression Tests

Uniaxial compression of the cylindrical gels (16 mm in diameter and 17 mm thick) was performed by means of a universal testing machine (Mecmesin Multitest 2.5-i) equipped with a 100 N load cell. The hydrogel disks were loaded onto the machine stage after removing the upper convex part with a blade and measuring their height using a calliper. A compression speed of 1 mm/min was used to deform the hydrogels to up to 15% of their initial height and return. The hysteresis area, calculated from the stress-strain graphs, was considered as a parameter to compare the hydrogels. The deformation range in which a linear stress-strain response is detected, for all hydrogels, was up to 5%. No liquid loss from hydrogels was detected during the compression experiments.

### 4.3. Rheological Characterization

Rheological characterization of the hydrogel disks (20 mm in diameter and 2–2.5 mm thick) was performed by means of HAAKE MARS III rheometer (ThermoScientific, Waltham, MA, USA) operating at 37 °C with cross-hatched parallel plate configuration. During the measurements, a glass bell was used as a solvent trap to prevent water evaporation from the hydrogels. To prevent excessive hydrogel squeezing, the gap between plates was adjusted for every hydrogel tested by performing a series of short stress sweep tests (𝜈 = 1 Hz; stress range 1–5 Pa) until a constant elastic modulus *G′* was reached. The linear viscoelastic range was determined by means of stress sweep tests consisting in measuring the elastic (*G′*) and viscous (*G″*) moduli variation with increasing shear stress (1 Pa < *τ* < 1000 Pa) at a frequency 𝜈 = 1 Hz.

### 4.4. BSA Diffusion Tests

The hydrogels were prepared in the presence of deionized water, LiCl 0.5 M or NaCl 0.5 M as solvent, respectively. The upper convex part of the cylindrical hydrogels (16 mm in diameter and 17 mm thick) was removed with a blade prior to placing every hydrogel in a 50 mL tube containing 10 mL of the respective solvent (deionized water, LiCl 0.5 M, and NaCl 0.5 M). The tubes were kept in a shaker at 37 °C for the entire duration of the test. At each time point (10 min, 20 min, 30 min, 1 h, 2 h, 4 h, 6 h), 2 mL of the solvent was removed from the tube and replaced with 2 mL of pure solvent. The removed volume was placed in a quartz cuvette to measure the absorbance at 280 nm using a spectrophotometer (Ultraspec 2100 pro, Amersham Bioscience, Amersham, UK). The BSA concentration was determined from the calibration curves prepared for each solvent used. The release of BSA in the solvent was evaluated as a function of time. BSA diffusion coefficient was calculated by data fitting according to the two dimensional mass balance embodying Fick’s (Equation (6)) law:

(6) $$\frac{\partial C}{\partial t}=\frac{D}{R}\frac{\partial }{\partial R}\left(R\frac{\partial C}{\partial R}\right)+D\frac{\partial^{2}C}{\partial Z^{2}}$$

where *D* (cm^2^/s) is the BSA diffusion coefficient in the hydrogel, *t* is the time, *C* is the protein concentration (mass/volume) in the hydrogel, *R* and *Z* are the radial and axial axes, respectively. Due to the uniform BSA loading inside the hydrogel, no tangential diffusion can occur so that the intrinsic three-dimensional diffusion problem reduces to a simpler two-dimensional one. The boundary conditions are defined as (Equation (7)):

(7) $$C\left(Z,R,t\right)=C_{tot},\;\;0\leq Z\leq Z_{c},\;\;0\leq R\leq R_{c},\;\;C_{out}\left(t=0\right)=0$$

where *Z_c_* and *R_c_* are the hydrogel height and radius, respectively, whereas *C_tot_* is the initial BSA concentration in the hydrogel while *C_out_* is the concentration of BSA in the release medium. The protein present in the reservoir at each time point is calculated as (Equation (8)) that represents the BSA mass balance on the hydrogel—release medium system:

(8) $$V_{r}C_{out}\left(t\right)=\pi {R_{c}}^{2}Z_{c}C_{tot}-\int_{0}^{Z_{c}}\int_{0}^{R_{c}}C_{tot}\left(Z,R,t\right)2\pi RdRdZ$$

where *V_r_* is the volume of the release medium. Data fitting was performed minimizing the sum of the squared errors between experimental data and Equation (6), assuming *D* as fitting parameter.

### 4.5. Low Field NMR Relaxometry

Low field NMR characterization on agarose hydrogels obtained in the presence of different amount of supporting salt was performed by means of a Bruker Minispec mq20 (static magnetic field *B*_0_ = 0.47 T) operating at 25 °C. Transverse relaxation time (*T*_2_) measurements were performed according to CPMG (Carr-Purcell-Meiboom-Gill) sequence with a 90–180° pulse separation of 0.25 ms (number of scans = 4; delay = 10 s). *T*_2_ was determined by fitting the experimental time (*t*) decay of the magnetization vector on the x-y plane (M_xy_) (Equation (9)):

(9) $$\frac{M_{\mathrm{xy}}}{M_{\mathrm{xy}0}}=\sum_{i=1}^{N}A_{i}e^{\left\{-\frac{t}{T_{2i}}\right\}}$$

where *M*_xy0_ is the initial *M*_xy_ value, *t* is time, *T*_2i_ are the relaxation times whose relative abundances are represented by *A*_i_. Equation (9) fitting to experimental relaxation data reveals that only one (*N* = 1) exponential term is statistically necessary. Thus, we are dealing with homogeneous hydrogels as all water molecules relaxes in the same way. Agarose solution was poured into the NMR holder (Ø = 8 mm) to fill it for about 2 cm from its bottom and the temperature was decreased to allow hydrogel formation. According to [42], the relation between the inverse of the experimental relaxation time ( ${\left(\frac{1}{T_{2}}\right)}_{\exp}$ ), the free water relaxation time (*T*_2free_), the average mesh size ( $\bar{\xi }$ ), the polymer volume fraction ν_p_ (=6.6 × 10^−2^) and the relaxivity M is given by

(10) $${\left(\frac{1}{T_{2}}\right)}_{\exp}=\frac{1}{T_{2\mathrm{free}}}+M\frac{2}{\bar{\xi }\sqrt{\;\;\frac{C_{0}}{C_{1}}\;\;\frac{1\;-\;0{.58\nu }_{p}}{\nu_{p}}}}$$

where *C*_0_ and *C*_1_ are two constants depending on network shape (for a cubical network, *C*_0_ = 1 and *C*_1_ = 3π) and M represents the effect of the polymer chains surface on water protons relaxation. Indeed, M is equal to the ratio between the thickness (*a*) and the relaxation time (*T*_2b_) of the bound water layer coating the chains surface:

(11) $$M=\frac{a}{T_{2b}}=\frac{R_{c}-R_{f}}{T_{2b}}$$

where *R*_f_ is the polymer chain radius and *R*_c_ is the radius defining the radial limit of the bound water layer. According to [42,45], *R*_f_ and *R*_c_ can be expressed, respectively, by:

(12) $$R_{f}=\frac{\bar{\xi }}{\sqrt{\;\;\frac{C_{1}}{C_{0}}\;\;\frac{1\;-\;0{.58\nu }_{p}}{\nu_{p}}}}\;\;\;R_{c}=\frac{R_{f}}{\sqrt{\nu_{p}}}$$

Solving Equation (10) for M and equating the resulting M expression to Equation (11), in the light of Equation (12), we get:

(13) $$T_{2b}=\frac{2}{\left({\left(\frac{1}{T_{2}}\right)}_{\exp}-\frac{1}{T_{2\mathrm{free}}}\right)}\frac{1}{\frac{1\;-\;0{.58\nu }_{p}}{\nu_{p}}}\left(\frac{1-\sqrt{\nu_{p}}}{\sqrt{\nu_{p}}}\right)$$

(14) $$a=R_{c}-R_{f}=\frac{\bar{\xi }}{\sqrt{\;\;\frac{C_{1}}{C_{0}}\;\;\frac{1\;-\;0{.58\nu }_{p}}{\nu_{p}}}}\left(\frac{1-\sqrt{\nu_{p}}}{\sqrt{\nu_{p}}}\right)$$

Notably, *T*_2b_ does not depend on any other physical parameters (such as, for example, *R*_f_) other than ν_p_, *T*_2free_ and ${\left(\frac{1}{T_{2}}\right)}_{\exp}$ , i.e., physical parameters that are easily experimentally determinable. In addition, *T*_2b_, reasonably, does not depend on network architecture (constant *C*_0_ and *C*_1_ depend on the cubical, tetrahedral or octahedral network arrangement). On the contrary, *a* depends on both network architecture and average mesh size ( $\bar{\xi }$ ). As a matter of fact, Equations (13) and (14) allow to get information at the nano-level resorting to physical parameters that can be macroscopically determined.

### 4.6. Average Mesh Size Determination

According to Flory’s theory [46], the polymeric network crosslink density ρ_x_ can be determined from the elastic shear modulus $G_{0}$ (see Table 1):

(15) $$\rho_{x}=\left(\frac{G_{0}}{R\;T}\right)$$

where *R* is the universal gas constant and *T* is absolute temperature. The link between the crosslink density and the average mesh size, $\bar{\xi }$ , is established by the equivalent network theory [47]. Starting from the evidence that, in most cases, a detailed description of a real polymeric network is rather complicated, if not impossible, this theory suggests replacing the real network topology by and idealized one, sharing the same average ρ_x_. The idealized network is made up by a collection of identical spheres centred around each crosslink. Remembering the definition of crosslink density (moles of crosslinks per hydrogel unit volume), it turns out that the volume competing to each crosslink in the real network (1/(*N*_A_ρ_x_); *N*_A_ is the Avogadro number) equals the volume of each sphere as the two networks share the same ρ_x_. Thus, the relation between $\bar{\xi }$ and ρ_x_ reads:

(16) $$\frac{4}{3}\pi {\left(\frac{\bar{\xi }}{2}\right)}^{3}=\frac{1}{\rho_{x}N_{A}}===>\;\bar{\xi }=\sqrt[{3}]{6/{(\pi \rho }_{x}N_{A})}$$

Relying on Equations (15) and (16) it was possible to evaluate the average nesh size for all of the samples studied as reported in Table 2:

## Figures and Tables

**Table 1:** $G_{0}$ and $\gamma_{c}$ values obtained from Equations (1) and (2) in the main manuscript.

I (M)	LiCl		NaCl	
	$G_{0}$ (kPa)	$\gamma_{c}$ (%)	$G_{0}$ (kPa)	$\gamma_{c}$ (%)
0	9.1 ± 0.4	0.7 ± 0.4	9.1 ± 0.4	0.7 ± 0.4
0.15	8.6 ± 0.6	1.5 ± 0.7	9.4 ± 0.9	1.3 ± 0.9
0.30	8.4 ± 0.7	1.7 ± 0.8	8.6 ± 1.1	2.3 ± 1.3
0.50	7.8 ± 0.3	2.6 ± 0.8	8.4 ± 0.7	3.2 ± 1.1
0.75	6.8 ± 0.4	4.3 ± 0.6	7.3 ± 0.9	4.9 ± 0.8
1.00	6.8 ± 0.5	3.7 ± 1.0	9.4 ± 0.8	2.6 ± 0.2

**Table 2:** Crosslink density (ρ_x_) and average mesh size ( $\bar{\xi }$ ) referring to the different samples considered in this work. ρ_x_ and $\bar{\xi }$ have been evaluated according to Equations (15) and (16), respectively. Shear modulus ( $G_{0}$ ) values are reported in Table 1.

I (M)	LiCl		NaCl	
	ρ_x_ (mol/m^3^)	$\bar{\xi }$ (nm)	ρ_x_ (mol/m^3^)	$\bar{\xi }$ (nm)
0	3.53	9.66	3.53	9.66
0.15	3.34	9.84	3.65	9.56
0.30	3.24	9.94	3.34	9.84
0.50	3.02	10.18	3.28	9.91
0.75	2.65	10.63	2.84	10.38
1.00	2.63	10.66	3.65	9.55

## Data Availability

Raw data generated during this study are available upon reasonable request. Correspondence and requests for materials should be addressed to P. Sacco.

## Supplementary Materials

The following supporting information can be downloaded at: www.mdpi.com/xxx/s1. Figure S1: Shear modulus (a) and critical strain (b) for 1 % (*w*/*V*) agarose hydrogels prepared in LiCl 1M and washed for 24 h in water or LiCl 1M; Figure S2: Concentration profile of BSA inside agarose hydrogels at time 0 (a) and after 15 min (b), 30 min (c) and 60 min (d) of the diffusion in the presence of NaCl 0.5 M in the external reservoir. Calculations performed following Fick’s second law (Equation (6)); Figure S3: Concentration profile of BSA inside agarose hydrogels at time 0 (a) and after 15 min (b), 30 min (c) and 60 min (d) of the diffusion in the presence of water in the external reservoir. Calculations performed following Fick’s second law (Equation (6)); Table S1: Diffusion coefficient, *D*, for BSA in agarose hydrogels prepared in different conditions evaluated by fitting experimental data with Fick’s second law (Equation (6)).
