# Supplementary material for: Ionic Strength Impacts the Physical Properties of Agarose Hydrogels
Source: Gels. 2024 Jan 25;10(2):0. doi: 10.3390/gels10020094 (PMC11154414; doi:10.3390/gels10020094)
Supplement: Supplementary file 1 [file gels-10-00094-s001.zip › gels-2799010-supplementary.pdf]

Supporting Information

Figure S1

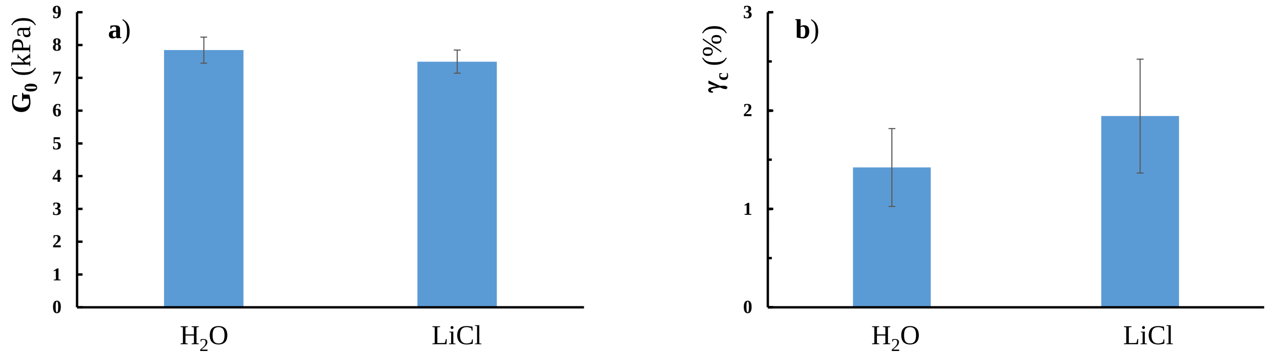

Figure S1. Shear modulus (a) and critical strain (b) for 1 % (w/V) agarose hydrogels prepared in LiCl 1M and whased for 24 h in water or LiCl 1M.

Table S1. Diffusion coefficient, D, for BSA in agarose hydrogels prepared in different conditions evaluated by fitting experimental data with Fick's second law (eq. 3).

**Condition**	**D** (cm^2/s)
Water	$7.5 \cdot 10^{-7}$
NaCl 0.5M	$1.1 \cdot 10^{-7}$
LiCl 0.5M	$1.3 \cdot 10^{-7}$

Figure S2

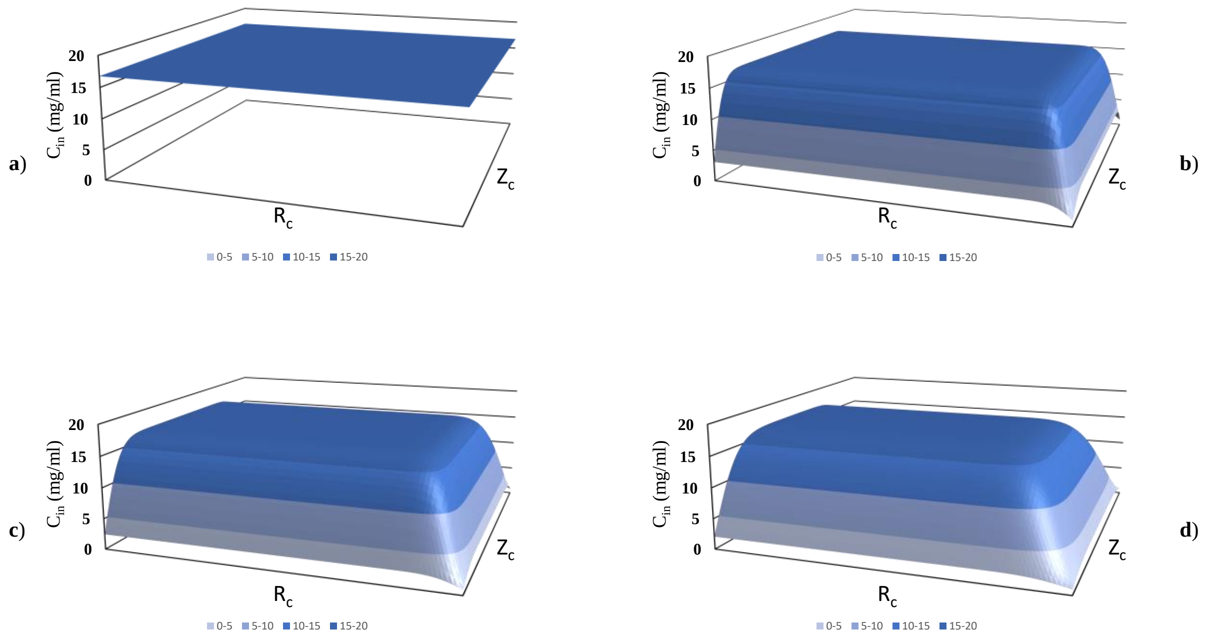

Figure S2. Concentration profile of BSA inside agarose hydrogels at time 0 (**a**) and after 15 min (**b**), 30 min (**c**) and 60 min (**d**) of the diffusion in the presence of NaCl 0.5 M in the external reservoir. Calculations performed following Fick’s second law (eq. 3).

Figure S3

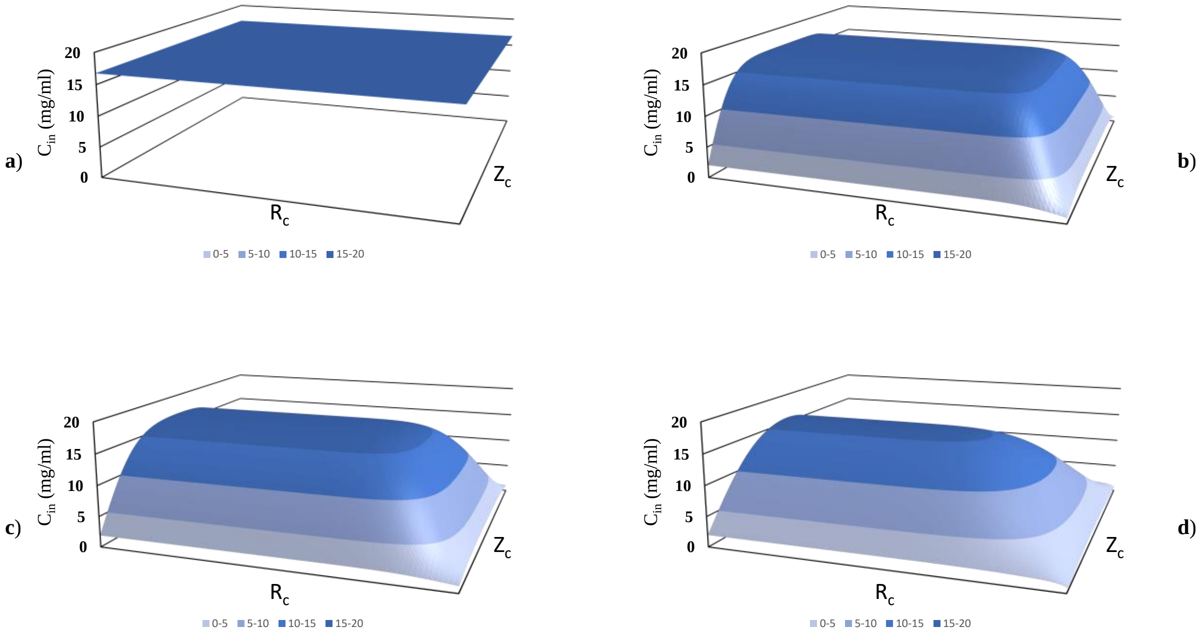

Figure S3. Concentration profile of BSA inside agarose hydrogels at time 0 (**a**) and after 15 min (**b**), 30 min (**c**) and 60 min (**d**) of the diffusion in the presence of water in the external reservoir. Calculations performed following Fick's second law (eq. 3).
